# Issues on the Scalp in Chronic Cerebral Diseases

**Published:** 1846-05

**Authors:** 


*Issues on the Scalp in Chronic Cerebral Diseases.*—The establishment of a caustic drain on the scalp over the suspected seat of the lesion, in cases of chronic cerebral disease, has proved in many hands a remedy of decided utility. I remember the case of a woman who suffered the most agonizing pain, notwithstanding the use of the most powerful narcotics, in consequence of the carious destruction of the petrous portion of the left temporal bone: the patient was much emaciated, and was affected with hectic fever. A potential cautery was applied over the squamous plate of the bone. As soon as the cauterized skin began to discharge, the relief was most gratifying; and the progress of the fatal event was very materially retarded. Women, who have, at some former period of life, suffered from a chronic irritation of the cerebral meninges, are apt to be affected, as life advances, with a most severe pain on the crown of the head, either along the course of the sagittal suture or a little to one side (usually to the right) of it. When such a case proves fatal, the cerebral meninges are generally found to be thickened and agglutinated together, and the glandulæ Pacchioni to be enlarged; this increase of size being sometimes so great as to have caused a considerable absorption of the bone over them. In such cases, all opiates and anti-spasmodics are utterly useless, and perhaps decidedly injurious: the only safe and judicious remedies are repeated local bleedings, and the establishment of an issue over the seat of the lesion.

For exciting the irritability of paralysed muscles, the most important of all remedies is unquestionably *electro-Magnetism*. I have seen many remarkable cures effected by its use. Not a few cases of paralysis of the generative organs have been speedily and effectually benefited by it. *Strychnine*, also, will be found to have very remarkable powers in curing certain forms of paralytic weakness. Its employment requires much discrimination and judgment; for, if administered too early, before the cerebral lesion has been very materially modified, mischief, and not benefit, will be the result. The same remark is applicable to the use of electricity.—*Ibid.*

